# Effectiveness of implementation of “mental health nursing students’ clinical competency model” on academic performance of nursing students

**DOI:** 10.12688/f1000research.14284.2

**Published:** 2018-11-17

**Authors:** Foroozan Atashzadeh-Shoorideh, Jamileh Mohtashami, Seyed Amir Hosein Pishgooie, Tayebeh Jamshidi, Sara Sedghi

**Affiliations:** 1 Department of Psychiatric Nursing, School of Nursing and Midwifery, Shahid Beheshti University of Medical Sciences, Tehran, 1996835119, Iran; 2 Faculty of Nursing, AJA University of Medical Sciences, Tehran, 1615116139, Iran; 3 Department of Psychiatric Nursing, School of Nursing and Midwifery, Shahid Beheshti University of Medical Sciences, Tehran, 1996835119, Iran; 4 School of Nursing and Midwifery, Shahid Beheshti University of Medical Sciences, Tehran, 1996835119, Iran

**Keywords:** Clinical competency, Nursing, pattern, academic performance

## Abstract

**Background: **Clinical nursing competence in mental health is one of the most important topics in theoretical and practical nursing training with many factors affecting it. The purpose of this study is to determine the impact of the implementation of the “mental health nursing students’ clinical competence model” on nursing students’ academic performance.

**Methods**: This study is a semi experimental following one group of student nurses. “mental health nursing students’ clinical competence model” for undergraduate nursing student’s education was applied. The study population included 50 nursing students, who were studying from fifth semester to seventh semester and selected through census sampling. During the seventh semester after the completion of theoretical and practical courses in mental health nursing, re-evaluation was conducted and the scores before and after the implementation of the clinical competence model were compared.

**Results: **Rate of clinical competency before the intervention, was estimated at the level of non-mastered; and after intervention was at the level of mastered, demonstrating a significant difference (p<0.001). Areas of clinical competency scores before and after the intervention were compared which showed significant difference in all the areas except the mental competency areas (p<0.05).

**Conclusions:** The implementation of the “mental health nursing students’ clinical competence model” and appropriate planning for achievement of mental health nursing specialized competency can ensure the achievement of clinical competency by nursing students.

## Introduction

The purpose of nursing education is to transfer knowledge and help students, gain insights and skills necessary for nursing care ^1^. One of the important aims of universities and higher education centers of medical science is building capacity and skills in students, as well as to prepare them for health services and to provide health care to all members of the community ^2– 4^. A survey of one study of nursing education courses showed that abilities acquired by students were far from optimal, and they have not gained the skills and abilities necessary at the end of their training ^5^. It seems, education plays an important role in the development of professional nursing skills and it provides opportunities to gain a wide range of knowledge, problem solving abilities and critical thinking ^6^. Previous studies on novice nurses also suggest that in the transition from student to professional role, students have experienced a lack of preparation. Stressful experiences in novice nurses during this period are often associated with a lack of necessary skills in nursing practices and the lack of comparability between undergraduate education in universities and the situation in the workplace ^7^. However, Manoochehri *et al.* ^8, 9^, concluded that student employment after graduation can help.

Nurses’ knowledge and competence are based on their knowledge and the curriculum that is taught in universities. The training program is very important in gaining nursing values and achieving educational goals ^10^. This knowledge and the skills acquired are valuable, and have a direct effect on the student’s future career, and immediately after graduation when they start work ^11, 12^.

Nursing education programs with an emphasis on skill development provide an opportunity to increase student competence and adaptability to meet the clinical needs of novice nurses entering the workplace ^13^. Some studies have shown that there is a direct relationship between the level of clinical competence and the ability to apply their skills. In other words, a nurse who has higher competency, can take advantage of their skills in clinical practice ^14^. Studies have shown that novice nurses are not adequately prepared to deal with challenges in the work place ^15– 18^, nursing undergraduate curriculum cannot prepare students adequately for independent performance ^19^, and therefore they may have limited technological skills ^20^. One study found that nearly 49 percent of newly graduated nurses were involved in errors, indicating the gap between meeting minimum standards of practice and professional competence ^21^.

Competence is a complex and ambiguous concept and one of the more controversial issues in the nursing standards in various fields including education, clinical and management ^22^. Competencies comprise of different aspects of learning including knowledge, skills and attitudes. According to this definition, people should be able to fulfill their role or set of tasks to an appropriate level ^5^. The Australian Nursing and Midwifery Council (2005) defines competence as a combination of skills, knowledge, attitudes, values and abilities that underlies effective and high professional performance in a professional career area ^23, 24^.

Therefore, nurse educators are trying to design competency-based training programs. Competencies are built on scientific knowledge, but their development requires several activities, the most significant of which is the application of theoretical content to real life situations ^25^. In the curriculum and teaching-learning processes where a competency-based approach is used the program is based on a set of skills that every student should master ^26, 27^.

In reviews of competency in nursing, there are challenges which reflect the difficulties and complexity of the profession. Mental health nursing for various reasons, including lack of clarity about roles within mental health nursing and lack of standards for mental health care, is faced with many challenges. The Nurses Association of America defines psychiatric nursing (Mental Health Nursing) as the diagnosis and treatment of human responses to actual and potential mental health problems. The psychiatric mental health nursing identifies with aspects of care such as communication ^28^. Mental health nurses are nurse practitioners who show competencies, knowledge, skills and special abilities to care for people with mental health problems and mental disorders ^29^. The nature of mental health nursing, like all areas of care, is undergoing profound changes. These changes include dealing with an aging population, increase in variation in cultures, case management, changes in care situation (such as from hospital to community), competition in care patterns, maintaining employment opportunities, providing the necessary training for career development, new science and information technology, and finally the work of mental health nursing practice ^30, 31^.

In relation to the lack of adequate mental health content in nursing curricula prior to employment, there are concerns which led it being alleged that new graduate nurses were not adequately prepared to care for patients with mental disorders. These concerns could have significant effects on the standards of care provided ^32, 33^. In a study that was conducted by Melnyck and colleagues at Yale University, officials believed that nearly half of new graduates were impaired when it came to providing comprehensive advice, especially to families at risk ^34^. There are also growing concerns about mental health nurses, in that they have not been adequately trained in medication management. This problem is even present in countries such as Britain, which since 2003 has given the right to prescribe medication to mental health nurses ^35^.

In Iran, the necessity of clinical competence has become more of an important issue in recent years, as communities now expect a higher quality of service, forcing health care system to increase the effectiveness of their staff ^22^. Despite the extraordinary importance of the topic, understanding of nursing clinical competency and skill level is limited and little research has been done in this area ^14^. Clinical competence requires a framework that includes access to all essential competencies during students’ education. This model means that all the requirements of clinical competence of nursing students needs be considered. Planning a curriculum with respect to all issues of clinical competence may be possible. A model for “mental health nursing students’ clinical competence” is a guide which provides a framework for achieving educational goals, performing evaluations and also allowing students to develop appropriate professional experience. Such a model helps establish and provide clinical competency-based planning. The aim of this study was to determine the impact of the implementation of ‘mental health nursing students’ clinical competence model’ on nursing students’ academic performance.

## Methods

This study is a semi experimental study conducted from January 2014 to January 2017. The sample was one group of undergraduate nursing students followed from fifth till their seventh semester. During three semester (18 months) students were taught based on the ‘mental health nursing students’ clinical competence model’ ^36^ in theoretical and clinical programs. The study population included 50 nursing students of Shahid Beheshti University of Medical Sciences, who were been selected through census sampling.

First, using the “mental health nursing students’ clinical competencies” checklist ( Supplementary File 1), designed in 2014 by Mohtashami *et al*. ^37^ pre-tests were conducted among 5 ^th^ semester nursing students who were taking the mental health nursing course. The checklist includes 73 statements which assess clinical competencies in two areas of general competency (emotional competency, 7 statements; moral competency, 11 statement; general nursing skill competency, 7 statements) and specific competencies (therapeutic communication competency, 12 statements and caring for mentally ill patient skill competency, 36 statements) using a Likert scale of 5 from “always” to “never”. Clinical competency were divided into 4 categories of weak (scores from 73 to 146), Average (scores from 146 to 219), good (scores from 219 to 292) and Excellent (scores from 292 to 365). Regarding to determination of mastery, we considered a score of 300 as the cutoff point, with scores below 300 indicating lack of mastery and scores above 300 showing competencies having been mastered. Reliability coefficient was conducted through internal consistency reliability (Cronbach’s alpha 0.93). The meaning of academic performance in this study was the final score that student got from mental health nursing course.

**Figure 1.**
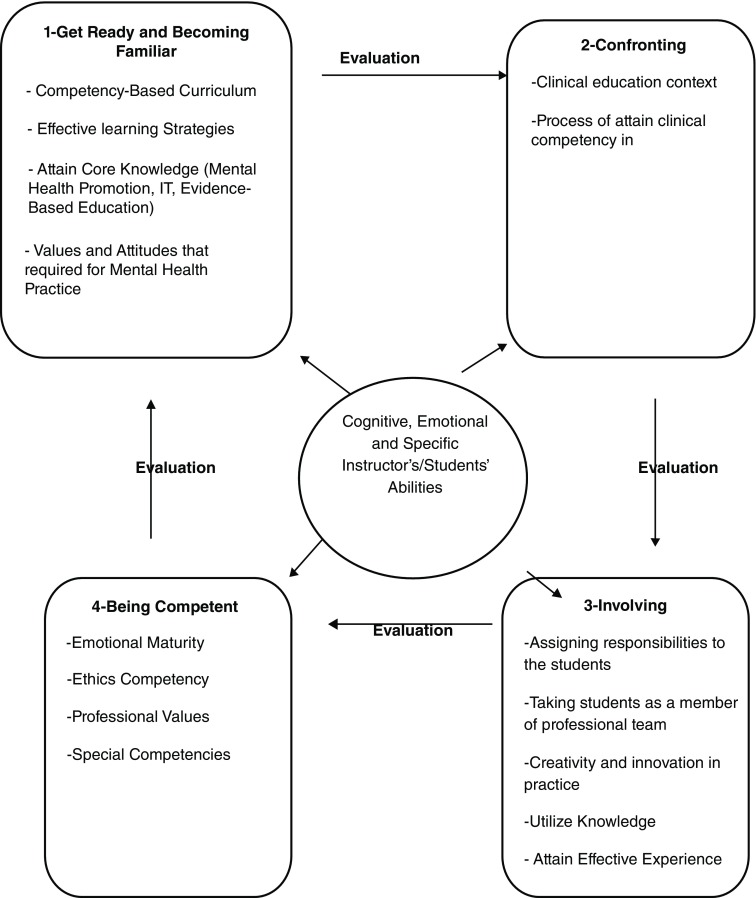
Mental health nursing students’ clinical competency model.

After the pre-test, the ‘mental health nursing students’ clinical competence model’ ^36^ was conducted. The hypothesis of this research was this model can improve mental health nursing clinical competency in students. The model has 4 related phases which started from a mental health nursing course (fifth semester) to apprentice in clinical field (seventh semester). In each phrase, every student is required to gain the knowledge, skills and special abilities of that phase. When passing through each phase, competency-based assessments were conducted. In the fifth semester where all students are studying mental health nursing, implementation of these models began. Later in the 6 ^th^ semester, psychiatric disorders and mental health nursing course were completed. Internships were also undertaken in this semester. Finally the intervention was completed in the 7 ^th^ semester after the apprenticeship in clinical field. The aim of this model is to achieve clinical competency through a systematic and scientific process and continuously be developed these competencies. Model dimensions were considered in four domains of orientation and preparation, confrontation, involvement and achieving clinical competency ( Figure 1). A breakdown of teaching aims for each domain is provided in Supplementary File 2.

In all domains of the model, cognitive/emotional abilities and specific skills of instructors and students will have a certain impact on clinical competency achievement. It should be noted that while transitioning from one stage to the next one, students must be evaluated to ensure achievement of required competencies of that stage.

Upon completion of the theoretical and clinical mental health nursing courses in the 7 ^th^ semester, re-evaluation was performed; and scores of before and after the implementation of the clinical competency model were compared.

The study was approved by the Ethics Committee of the School of Nursing in July 2015 with approval number SBMU2.REC.2015.6. All the ethical considerations were taken into account. All the participants signed a consent form before participating in the study.

In this study, data analysis was conducted through the use of SPSS software-version 21. First, to describe the demographic characteristics of subjects, review frequency distribution, mean and standard deviation using descriptive statistical tests, were used. In the following, Paired t-test analysis, the nonparametric equivalents of it depending on the type and distribution of data, were used. The significance level for all tests was set at 0.05.

## Results

50 nursing students were enrolled in this study. Most (90%), were aged 21 to 25 years old (N=45) 62 percent (N=31) were women and 38 percent (N=19) were male. Statistical analysis of the relationship between age and gender and the clinical competencies did not find any significant relationship. Average scores in different domains of clinical competency before and after the implementation of competence model are shown in Table 1. Rate of clinical competency prior to the implementation was estimated in the level of the non-mastered and after the implementation was in the level of mastered, a statistically significant difference ( Table 1). Clinical competency areas using paired t-test scores were compared before and after the intervention. All scores before and after treatment in all areas except the area of mental competencies, showed a significant difference (p <0.05).

**Table 1.** Mental health nursing students’ clinical competencies before and after the intervention.

Clinical Competencies Issues	Score	Before intervention		After intervention		The mean difference before and after intervention	The standard deviation difference before and after intervention	Paired t test
		Mean	standard deviation	Mean	standard deviation			
Emotional	7–35	31.68	3.74	35.30	2.03	3.62	3.06	t=8.35 p<0.001
Ethical	11–55	41.92	4.18	45.30	2.42	3.83	3.26	t=7.32 p<0.001
General skills	7–35	27.36	3.96	30.84	2.03	3.48	2.80	t=8.76 p<0.001
**General** **competency**	25–125	100.96	10.11	111.44	4.90	3.49	2.52	t=9.79 p<0.001
Therapeutic communication skills	12–60	52.66	9.37	55.04	3.24	2.38	8.98	t=1.87 p<0.001
Skill of care for patients with psychiatric disorders	36–180	145.96	18.03	159.36	10.19	13.40	10.58	t=8.95 p<0.001
**Specific** **Competencies**	48–240	198.62	22.81	214.4	12.75	7.89	1.05	t=7.53 p<0.001
Total	73–365	299.58(non- mastered)	30.85	325.84 (mastered)	44.86	325.84	16.38	t=8.97 p<0.001

Test scores pre and post intervention with demographic information Click here for additional data file. Copyright: © 2018 Atashzadeh-Shoorideh F et al. 2018 Data associated with the article are available under the terms of the Creative Commons Zero "No rights reserved" data waiver (CC0 1.0 Public domain dedication).

## Discussion

Clinical competence requires a framework that includes all aspects of access to essential competencies during students’ education. This model means that all the requirements of clinical competence of nursing students need to be considered, and appropriate curriculum with respect to all issues related to clinical competence is useful. Existence of a model for mental health nursing students’ clinical competence, in addition to determine the achievement of educational goals, provides the possibility to evaluate and gain feedback; it also provides the opportunity for reformation of professional practice for nursing students. Such a model helps establish and provide clinical competency-based planning. The aim of this study was to evaluate the effects of the ‘mental health nursing students’ clinical competence model’ on nursing students’ academic performance.

This research findings showed that rate of clinical competency before the intervention, was estimated at the level of non-mastered; and after intervention was at the level of mastered, this difference was statistically significant. Wangensteen *et al*. ^38^ in relation to newly graduated nurses’ perception of competence and possible predictors also found the same results. In line with the current study, Safadi *et al*. ^39^ conducted a cross-sectional study to reviews competencies of nursing graduates in universities in Jordan. The results were rated as satisfactory clinical competence in line with this research Mohtashami *et al.* ^40^ in the article appearing with the aim of determining the relationship between professional competence and professional identity, concluded that there is a positive correlation between professional competencies and professional identity.

The results of a cross-sectional study by Salonen *et al*. ^41^ in relation to the clinical competency of Finnish novice nurse’s and factors affecting it showed, fair to good level of clinical competence which was different from the findings of the current study. Reasons for these differences may include using a different instrument, the study samples and that the nurse’s had a higher level of experiences. Phillips *et al*. ^42^ in this regard reported that an increase in level of clinical experience and more hours in school and placement in the clinical setting, especially in nursing education programs, promote decision-making skills, more professional performance in individuals and gaining teamwork experience in the real working environment. Boru Bifftu *et al.* found that overall, 48.7% of the study participants perceived themselves as having high clinical competence ^43^.

All scores before and after treatment in all areas except the area of mental competencies, showed a significant difference especially in skill of care for patients with psychiatric disorders (specific competencies) p<0.001. Bradshaw believe that “it is essential to develop a psychiatric nursing subject that not only teaches the student the necessary skills and knowledge to safely and effectively care for the mentally ill person” ^44^.

In a study by Sabancıogullari & Dogan ^45^ the effects of the professional identity development program on the professional identity, job satisfaction and burnout levels of registered nurses was conducted on 63 nurses working in a university hospital. The program of 10 sessions (once a week, and follow-up 6 months later) improved the professional identity of nurses in the intervention group compared to the control group which the difference was statistically significant. During the study period burnout among nurses in the intervention group decreased, but increased in the control group. But there was no statistically significant difference between the intervention group and the control group in terms of job satisfaction.

Mohtashami *et al.* ^46^ conducted a qualitative study aimed to clarify the concept and how to achieve clinical competencies in mental health nursing students, concluding that nursing students in undergraduate education in order to gain mental health nursing competencies, must pass special stages before being be able to work. During each stage there are indicators that can help students to acquire the competencies. Therefore changes in the curriculum and students training methods should be considered. In support of this claim, Mohtashami *et al*. ^47^ wrote a competency-based curriculum to facilitate the teaching-learning process is one of the first steps. Revision of curriculum can be used to reduce gap between theory and practice so competencies can be acquired effectively. Bradshaw reported that “one in five Australians is likely to experience a major mental illness at some time in their lives. It is imperative that nursing programs prepare graduates to care for this health consumer group through effective mental health nursing education“ ^44^.

In a literature review, the researchers could not find similar studies, therefore it can be argued this study aiming to influence the implementation of clinical competence of nursing students’ mental health and academic performance is in itself an innovation. On the other hand, no similar study was found to allow more comprehensive discussion possibility and that can be considered a limitation of this study.

## Implications for practice

The implementation of the ‘mental health nursing students’ clinical competence model’ and appropriate planning for achievement of mental health nursing specialized competency can ensure the achievement of clinical competency by nursing students. Such a model helps establish and provide clinical competency-based planning.

## Data availability

The data referenced by this article are under copyright with the following copyright statement: Copyright: © 2018 Atashzadeh-Shoorideh F et al.

Data associated with the article are available under the terms of the Creative Commons Zero "No rights reserved" data waiver (CC0 1.0 Public domain dedication). 

Dataset 1: Test scores pre and post intervention with demographic information 10.5256/f1000research.14284.d207075 ^48^

## Supplementary material

Supplementary File 1: “mental health nursing students’ clinical competencies” checklist.

Supplementary File 2: File contain expansion of study design including detailed explanation of teaching aims for each domain.
