# Supplementary material for: Effectiveness of implementation of “mental health nursing students’ clinical competency model” on academic performance of nursing students
Source: F1000Res. 2018 Nov 17;7:1212. Originally published 2018 Aug 7. [Version 2] doi: 10.12688/f1000research.14284.2 (PMC6249634; doi:10.12688/f1000research.14284.2)
Supplement: Supplementary file 2 [file f1000research-7-18577-s0001.tgz › 089116b6-083b-4b15-afa4-3be8b92c1af4.docx]

**Clinical Competency Evaluation in Mental Health Nurses Checklist**

| **Always**  **(4)** | **Mostly**  **(3)** | **Sometimes**  **(2)** | **Barely**  **(1)** | **Never**  **(0 )** | **Items** | **No.** |
| --- | --- | --- | --- | --- | --- | --- |
|  |  |  |  |  | **General Competency**  *Emotional Competency* |  |
|  |  |  |  |  | Utilize relationship communication with colleagues | 1 |
|  |  |  |  |  | Control emotional status in critical situation | 2 |
|  |  |  |  |  | Coping with problems | 3 |
|  |  |  |  |  | Evaluate her/his weakness and strengths | 4 |
|  |  |  |  |  | Control her/his negative attitudes towards psychiatric patients | 5 |
|  |  |  |  |  | Demonstrate interest and motivation in caring | 6 |
|  |  |  |  |  | Demonstrate positive attitude in professional identity | 7 |
|  |  |  |  |  | *Ethical Competency*  Demonstrate patience when taking care of patient | 8 |
|  |  |  |  |  | Consider towards psychiatric patient as valuable human | 9 |
|  |  |  |  |  | Avoid rushing into judgment about patient | 10 |
|  |  |  |  |  | Keep the secret’s patient(unless there is risk) | 11 |
|  |  |  |  |  | Consider patients’ rights (e.g. privacy ,refuse treatment,etc.) | 12 |
|  |  |  |  |  | Utilize ethical principle in caring(e.g. confidentiality, autonomy,etc.) | 13 |
|  |  |  |  |  | Provide nursing care organized | 14 |
|  |  |  |  |  | She/he is responsible | 15 |
|  |  |  |  |  | She/he is flexible | 16 |
|  |  |  |  |  | Respect to racial ,cultural and religious specifications | 17 |
|  |  |  |  |  | Provide nursing care based on patients’ cultural specifications | 18 |
|  |  |  |  |  | *General Skills*  Show clinical decision making ability | 19 |
|  |  |  |  |  | Have necessary action speed | 20 |
|  |  |  |  |  | Consider every change in environment | 21 |
|  |  |  |  |  | Utilize theoretical knowledge in practice | 22 |
|  |  |  |  |  | Utilize problem solving skills | 23 |
|  |  |  |  |  | Utilize practical skills (e.g. change dressing, injections, and control of vital signs ,etc.) | 24 |
|  |  |  |  |  | Provide tele nursing (e.g. internet,media,telephon, etc.) | 25 |
|  |  |  |  |  | **Specific Clinical Competency**  *Therapeutic Communication*  Provide the principles of interviewing from the beginning to the end | 26 |
|  |  |  |  |  | Gain the patient’s trust to establish communication | 27 |
|  |  |  |  |  | Motivate the patients to express their feelings | 28 |
|  |  |  |  |  | Observe the limits (physical distance, to address the patient with respectful titles, ) | 29 |
|  |  |  |  |  | Follow an active listening method in communication with the patient | 30 |
|  |  |  |  |  | Spend adequate time for communication | 31 |
|  |  |  |  |  | Avoid criticism and insults to the patient | 32 |
|  |  |  |  |  | Help the patient to make relationship with others | 33 |
|  |  |  |  |  | Help the patient to improve their self-confidence through improving capabilities and advantages of the  patient | 34 |
|  |  |  |  |  | Explain medical procedures before starting them (e.g. Injection, vital sign checkup, administering medicines,etc.)) | 35 |
|  |  |  |  |  | Use the medical procedures as a chance to communicate with the patient | 36 |
|  |  |  |  |  | Record and recognize defensive mechanisms used by the patients (e.g. projection, denial, displacement, etc.) | 37 |
|  |  |  |  |  | **Specific psychiatric care skills**  Observe the patient carefully to ensure that the patient has taken their medicine | 38 |
|  |  |  |  |  | Use nursing measures in the fields of using psych pharmacy drugs | 39 |
|  |  |  |  |  | Observe psych pharmacy drugs and the side-effects | 40 |
|  |  |  |  |  | Provide appropriate intervention in PRN medication(e,g. privacy, accurate dosage, etc.) | 41 |
|  |  |  |  |  | Use a variety of references (e.g. family, medical file, etc.) to collect information | 42 |
|  |  |  |  |  | Assess patient’s mental status examination frequently | 43 |
|  |  |  |  |  | Use different screening tools (narcotic drug abuse, personality disorder, depression, etc.) to examine  hazardous behavior (e.g. aggressiveness, suicide, murder, etc.) | 44 |
|  |  |  |  |  | Record the results of patient assessment and problems. | 45 |
|  |  |  |  |  | Prioritize psychiatric nursing diagnosis in providing health care to the patients | 46 |
|  |  |  |  |  | Record and report any change in the mental health of the patient and other problems | 47 |
|  |  |  |  |  | Carry out the principle of triage in emergency situation | 48 |
|  |  |  |  |  | Carry out the required health care in emergency and attack cases (e.g. physical restrain, isolation room, etc.)**.** | 49 |
|  |  |  |  |  | Provide a safe environment for the patient (by taking care of oneself, other, escaping, etc.) | 50 |
|  |  |  |  |  | Apply appropriate therapeutic procedure base on patient diagnosis | 51 |
|  |  |  |  |  | Auditing the quality of nursing care | 52 |
|  |  |  |  |  | Pay attention to patient’s needs (e.g. physical ,psychological ,social and spiritual) | 53 |
|  |  |  |  |  | Help to patient in self- care activity | 54 |
|  |  |  |  |  | Carry out nursing skills based on reliable references and new researches (evidence based( | 55 |
|  |  |  |  |  | Record any intervention accuracy | 56 |
|  |  |  |  |  | Monitor of consequences of patients’ care | 57 |
|  |  |  |  |  | Pay attention to optimum usage of medicines, materials, and equipment | 58 |
|  |  |  |  |  | Deliver appropriate patient’s report to other colleague | 59 |
|  |  |  |  |  | Cooperate and consult with other members of the health team | 60 |
|  |  |  |  |  | Have accountability about patient’s needs | 61 |
|  |  |  |  |  | Have accountability about family’s needs | 62 |
|  |  |  |  |  | To motivate the patient to follow the treatment program | 63 |
|  |  |  |  |  | Provide consultation services to patient and family | 64 |
|  |  |  |  |  | Encourage family to participate in patient care | 65 |
|  |  |  |  |  | To prepare discharge plan for the patient and their family | 66 |
|  |  |  |  |  | To follow psychological rehabilitation principles and standards (e.g. self-care, treatment follow up, etc.) to  improve patient’s health and to help them in regaining their abilities | 67 |
|  |  |  |  |  | Follow up discharge program | 68 |
|  |  |  |  |  | Provide required training to improve the patient’s health | 69 |
|  |  |  |  |  | Provide required training to improve the family’s health | 70 |
|  |  |  |  |  | Apply appropriate educational materials depend on patient’s characteristics | 71 |
|  |  |  |  |  | To use learning opportunities such as continuing studies, and participating in educational workshops to  improve personal and professional progress | 72 |
|  |  |  |  |  | Apply professional standards | 73 |
