# Supplementary material for: Effectiveness of implementation of “mental health nursing students’ clinical competency model” on academic performance of nursing students
Source: F1000Res. 2018 Nov 17;7:1212. Originally published 2018 Aug 7. [Version 2] doi: 10.12688/f1000research.14284.2 (PMC6249634; doi:10.12688/f1000research.14284.2)
Supplement: Supplementary file 3 [file f1000research-7-18577-s0002.tgz › 7454d060-983d-4cc7-a9f7-c88070f1a200.docx]

**Study Design and Methods**

Implementation of this model begun during the fifth semester while nursing students studied “Mental Health Nursing (1)” lesson. Following the first course; in the sixth semester, students continued on “Mental Health Nursing (2)” lesson and its internship, and in 7^th^ semester with apprenticeship in the field they completed the mental health curriculum.

In this model, clinical competency was achieved through a systematic and scientific process and continuously was developed. Model dimensions were considered in four domains of orientation and preparation, confrontation, involvement and achieving clinical competency (Figure 1.), which will be discus in following.

1. Orientation and Preparation

In this dimension, suggested model contain of:

- “Mental Health Nursing Students’ Clinical Competency Model” includes competency based curriculum, which has these characters:
  - Achieving clinical competence based on the principles and content of modern and functional curriculum is carried out.
  - A common language can be established between educational field and the clinical field.
  - The lesson plan, explanation and definition of mental health nurse competencies are presented.
  - The educational content is proportional to the role of mental health nursing.
  - Educational needs and gaps are identified in student clinical experiences.
  - Self-educating education is established and new approaches in education are used.
  - The attitude of the student is promoted during the training of competencies achievement.
  - Rewarding values and professional ethics for developing clinical competence will be promoted.

The curriculum, in the first dimension of the proposed model is included of theoretical lessons of individual and social psychology, Nursing of mental health(1) and Nursing in mental health(2) and internships.

Based on the findings of the first part of this study and the researchers experiences: inadequate theoretical hours is one of the main reasons for lack of preparation and achieving knowledge and specialized skills of graduates in the role of mental health nursing. Therefore in mental health nursing curriculum in this proposed model, there are changes such as increase in hours of theoretical courses of “Mental Health 1” from 2 courses to 3 courses, and of “Mental Health2” from 2 courses to 3 courses. Also due to the increased prevalence of mental disorders and lack of some important theoretical lessons in the current curriculum, Students' lack of familiarity with nursing profession in mental health and negative attitudes towards the profession, in this model the following topics are emphasized: psychiatric nursing and improving community attitudes toward this profession, different fields and positions in this profession (such as hospitals, schools and society) and comparison with other countries, legitimate and legal issues, understanding Psychological Tests and Screening for Mental Disorders, sleeping disorders, eating disorders and somatization disorders, A review of common disorders of child and adolescent psychology, Compliance with legal and ethical areas of psychiatric treatment.

Also, some studies have identified that there is a need for integration and increased hours of mental health internship is needed to achieve clinical competence.

Therefore, educators and students believed this will prevent the repetition of content in the first internship (which is provided in 6^th^ semester) in the final clinical internship. Students have adequate time to get acquainted with patients and obtain the necessary clinical competencies within a reasonable period of time. Thus, in the proposed model, two courses of internship which are currently presented in 6^th^ and 7^th^ semester, will be presented continually in 7^th^ and 8^th^ semester in 24 days.

- Effective learning strategies, which should pay attention to the followings:
- Educational goals in all three areas: cognitive, emotional, and psychomotor were designed.
- Different teaching methods such as feedback lecture, group discussion, demonstration, case study, role play, simulation; with the aim of acquiring skill and competence, are used.
- Self-directed learning is established by encouraging students to study individually, participating in in group discussions conferences, articles, teamwork, criticism of educational films, extending recognition through comparing cases, communicating with the patient, and providing continuous feedback to the student.
- Acquiring knowledge about:
  - Promoting mental health and preventing mental disorders (in every 3 stages of screening, early and proper diagnosis and psychological rehabilitation.
  - Types of psychiatric disorders, signs and symptoms, treatment and care
  - Use and application of information technology in mental health nursing care
  - Providing Biological, psychological, social and spiritual care
  - Values, attitudes and professional ethics required for practical work of mental health nursing

Evaluation would be in form of Quiz exams, mid-term, end-of-course, presentation of the conference, essay, group work, research work, educational film reviews.

1. Confronting

This has clinical education context and process of attain clinical competency in setting.

- The structure of clinical education for effective clinical education, Includes the following:
  - Appropriate human resources and organization for providing students with educational services.
  - Use of comprehensive clinical education such as psychiatric hospitals, schools, factories, psychological centers, addiction treatment centers ...
  - Hospital wards which allows patients with various types of psychiatric disorders to be admitted.
  - Educational facilities such as library, computer site, ...
- Clinical teaching content

The process of obtaining clinical competency will be influenced by the content and how the clinical education will be implemented. Here are some points to note:

- Prerequisites for entering psychiatric wards.
- Preparing students for encounter and practical work with patients with psychiatric disorders and the use of knowledge gained in practice.
- The relevance of the content of the curriculum based on the outline of the curriculum (such as familiarity with diagnostic and therapeutic methods, Using evidence-based education based on the latest clinical research, the use of educational and therapeutic programs in psychiatric departments) and implementing the teaching / learning process tailored to learner’s needs.
- Appropriate duration of clinical education
- Continuity of clinical education course
- Clinical teaching methods

Also, the implementation of clinical education programs with using the actual cases of patients in the department and effective teaching methods, it enhances students' ability to do practical work with patients with psychiatric disorders. The length of continuous internship (24 continuous days) will be taught to students in learning and their ability to communicate between theoretical and clinical courses.

To evaluate student’s practical ability, they have to pass internship duration for 24 days, every day for 8 hours in psychiatric wards, psychiatric clinics, and schools and on community level. To achieve specified clinical goals, the instructor continually monitors student’s clinical work. Student’s activities would be participating in discussion and interlocution, question and answer, evidence-based education, simulation, role play and Preparing a folder for work in clinical practice.

Evaluation can be a deep introduction of cases, who they work with during their internship. Evaluation tool are completion of patient examination forms, presentation of the nursing process and taking clinical examinations.

1. Involvement

In this stage, after gaining theoretical knowledge and clinical trainings, by being in a clinical environment, students directly confront with psychiatric patients (alone without instructor). Components of this domain include:

- Assigning responsibilities to the students:

Being responsible and accountable towards offered responsibilities is one of the important and influential components of the model are the involvement of the student in the process of obtaining the necessary competencies of the mental health nurse. In this stage:

- The student achieves the belief that she is responsible for taking care of her patient, therefor she will be accountable for her patient’s needs.
- Students will be more involved in their education process.
- Using teaching methods such as case study, makes the student responsible for all the tasks of the patient and engages them in the care process.
- Allowing the student to have authority and responsibility in perform the duties assigned to him or her.
- Being a member of treatment team:

Participation and teamwork, in order to engage the student as a member of the treatment team, plays an essential role at this stage. In this stage:

- Student interaction with treatment team members, will enhances student decision-making skills and problem solving skills, and increases their enthusiasm for patient care.
- Students as a member of treatment team, participates with others and facilitate the decision-making process.
- The student sees themselves as an influential person in the care of the patient.
- Responsiveness and accountability of students should increase.
- Creativity, innovation and prosperity:

In this stage students would show critical thinking, innovation and creativity in providing patient care. In this stage students:

- Using critical thinking, search to find the root causes of problems.
- Able in identification, assessing and decision making about important information about patient care.
- Affects patient care by creating new ideas.
- Practice based on an evidence-based framework and use of research results.
- A systematic vision is formed in the student.
- Utilize knowledge and attain effective experience

In this stage student:

- Would apply their knowledge on the patient's bedside.
- This will provide an effective and efficient experience for caring for people with psychiatric disorders.
- Will be able to rely on personal judgment and others in identifying problems.
- Before any intervention, with a thorough assessment, they could predict pre any reactions.
- They could apply their knowledge of practical exercises.

Evaluation would be contain of history taking, mental status examination, implementation and record of nursing process, evaluation with “Clinical Competency Evaluation in Mental Health Nurses Checklist”, presentation of a case study, pharmacology, psychiatric education of patients and their families, instructor’s observation of therapeutic communication and interview of patients with mental disorders, psychiatric wards head nurse and staff’s reports, presentation of community level visits reports (such as schools, home visits, addiction treatment centers and ….). Successful passing of this stage will provide the opportunity to enter the next stage.

1. Being competent

In the final stage of the model, students succeed to achieve general and specialized competency in mental health nursing and were able to do as their professional roles in the field of mental health nursing. This contains:

- Emotional maturity:

In this stage, students should achieve these abilities:

- Ability to socially communicate with treatment members.
- Ability of emotional control in relation to patients with psychiatric disorders and the problems and problems that result from it.
- Ability to change and control of their negative mentality towards patients with mental illness.
- Students will be able to coordinate their morale with the characteristics and conditions of psychiatric departments.
- The student gets the enthusiasm, passion and interest in taking care of the patient.
- Ethical competency:

In this stage of obtaining practical competency, students should:

- To accept the psychiatric patient as a respectable and worthwhile person.
- Supports the patient.
- Keeping the patients secrets.
- Showing patience and tolerance while working with psychiatric patients.
- Considering the patient’s condition, they will be given the right to accept or refuse the treatment.
- Consider ethical and legal issues of physical restraint and seclusion.
- Giving nursing care considering the patients cultural and ethnic features of psychiatric patients.
- Respect patient’s beliefs and costumes.
- Being aware of the application of legal / ethical practices related to patient care (such as malpractice, wergild, etc.).
- Being aware of protection of themselves from possible harms from patients.
- Commitment to professional values:

Students should gain the following abilities and knowledge:

- Understanding the differences of mental health nursing practices from other nursing specialties.
- Overcome the existing stigma of mental illness and patients and psychiatric nursing profession.
- Knows the future of the mental health care nursing profession and career.
- Uses any learning opportunities for their Individual / Professional Continuous Development.
- Keeps up to date their knowledge and information with massive changes in science and information technology.
- Detect and promote the Professional Performance Standards.
- Knows the different areas and roles of mental health nurse (in schools, society, practical sets, education, research and management).
- special competency

In the final stage of the Mental Health Nursing Student Clinical Competency model, students could attain skills and abilities that are necessary for a psychiatric nurse such as: therapeutic communication, Care-based recovery and patient-centered, education, support, Consultation; Safe and quality care, continuous improvement of care, bio-psycho social spiritual care, Evidence Based Nursing; Use of Information Technology in Nursing Care, Providing remote health services; Promoting mental health; participating and teamwork; Providing community services; fallow up treatment.

In all domains of achieving clinical competency, cognitive/emotional abilities and specific skills of teachers and student will have a certain impact on quality of clinical competency achievement. It should be considered that while transition from one stage to next one, students should be evaluated as a feedback process to ensure the achievement of required competencies and skills for the next stage.

Finally, to achieve Mental Health Nurse Competency, the student must pass a minimum score in courses and dedicated topics of 12 (The minimum passing grade based on the regulations of the Nursing and Midwifery Schools) to determine the proper level of competency.

**Figure1. Mental Health Nursing Students’ Clinical Competency Model**

**1-Get Ready and Becoming Familiar**

- Competency-Based Curriculum

- Effective learning Strategies

- Attain Core Knowledge (Mental Health Promotion ,IT, Evidence-Based Education)

- Values and Attitudes that required for Mental Health Practice

**2-Confronting**

-Clinical education context

-Process of attain clinical competency in

**Evaluation**

1. **Evaluation Evaluation**

**Evaluation Evaluation**

**3-Involving**

-Assigning responsibilities to the students

-Taking students as a member of professional team

-Creativity and innovation in practice

-Utilize Knowledge

- Attain Effective Experience

**4-Being Competent**

-Emotional Maturity

-Ethics Competency

-Professional Values

-Special Competencies

1. **Evaluation**

**Evaluation**
