# Supplementary material for: Effectiveness of implementation of “mental health nursing students’ clinical competency model” on academic performance of nursing students
Source: F1000Res. 2018 Nov 17;7:1212. Originally published 2018 Aug 7. [Version 2] doi: 10.12688/f1000research.14284.2 (PMC6249634; doi:10.12688/f1000research.14284.2)
Supplement: Peer review history

# Peer review: Referee response for version 2

**DOI:** 10.5256/f1000research.18577.r40770

**Published:** 2018-11-21

**Authors:** Julie Bradshaw

**Affiliations:** 1 Central Queensland University (CQUniversity), Rockhampton, Qld, Australia

Am happy with the changes.

I have read this submission. I believe that I have an appropriate level of expertise to confirm that it is of an acceptable scientific standard.

# Peer review: Referee response for version 1

**DOI:** 10.5256/f1000research.15545.r38804

**Published:** 2018-10-08

**Authors:** Julie Bradshaw

**Affiliations:** 1 Central Queensland University (CQUniversity), Rockhampton, Qld, Australia

There is a fundamental flaw in the research question - clinical competence improving academic performance. Clinical competence was improved, not necessarily academic performance. No other intervention was tested. It is not known whether this specific intervention improved competence specifically. We are left wondering, isn't any education on mental health nursing likely to improve competence? There seemed to be a number of assumptions made in the introduction. It seemed that the authors were mixing up clinical competency and critical thinking. This is obviously an international study. I would have liked to see an argument built around the deficits in nursing education in this country and why this tool was necessary.

I have read this submission. I believe that I have an appropriate level of expertise to state that I do not consider it to be of an acceptable scientific standard, for reasons outlined above.

# Response

**Published:** 2018-11-06

**Authors:** Jamileh Mohtashami

**Affiliations:** Shahid Beheshti University of Medical Sciences, Iran

Thanks for your patience. I would like to say we corrected some parts of article according to reviewer Julie Bradshaw:

- In introduction we explained about competency in my country. 
- We corrected some parts in methodology and statistical analysis. 
- Moreover we deleted one paragraph and added some articles for adequately support in conclusion.
- Finally, the references are corrected.

# Peer review: Referee response for version 1

**DOI:** 10.5256/f1000research.15545.r36903

**Published:** 2018-09-12

**Authors:** Fataneh Ghadirian

**Affiliations:** 1 Psychiatric Nursing Department, School of Nursing and Midwifery, Tehran University of Medical Sciences, Tehran, Iran

The study’s subject is one of the most important issues for mental health nursing in the world and especially in Iran and their authors clearly and accurately presented its background and rationales.

 The work is technically sound with sufficient details. It was better to have a brief history of used checklist due to varied competency indicators all over the world but this is not so serious.

 There are sufficient details of statistical analysis and the datasets are clearly described.  Although there is a sufficient discussion included the necessary details but the study has no final conclusion statement.  

 Finally, the study is valuable and approved.

I have read this submission. I believe that I have an appropriate level of expertise to confirm that it is of an acceptable scientific standard.
